# Determination of the Best Empiric Method to Quantify the Amplified Spontaneous Emission Threshold in Polymeric Active Waveguides

**DOI:** 10.3390/molecules25132992

**Published:** 2020-06-30

**Authors:** Stefania Milanese, Maria Luisa De Giorgi, Marco Anni

**Affiliations:** Dipartimento di Matematica e Fisica “Ennio De Giorgi”, Università del Salento, Via per Arnesano, 73100 Lecce, Italy; stefy_mil_94@hotmail.it (S.M.); marialuisa.degiorgi@unisalento.it (M.L.D.G.)

**Keywords:** conjugated polymers, Amplified Spontaneous Emission, optically pumped laser, optical gain, active waveguides

## Abstract

Amplified Spontaneous Emission (ASE) threshold represents a crucial parameter often used to establish if a material is a good candidate for applications to lasers. Even if the ASE properties of conjugated polymers have been widely investigated, the specific literature is characterized by several methods to determine the ASE threshold, making comparison among the obtained values impossible. We quantitatively compare 9 different methods employed in literature to determine the ASE threshold, in order to find out the best candidate to determine the most accurate estimate of it. The experiment has been performed on thin films of an homopolymer, a copolymer and a host:guest polymer blend, namely poly(9,9-dioctylfluorene) (PFO), poly(9,9-dioctylfluorene-cobenzothiadiazole) (F8BT) and F8BT:poly(3- hexylthiophene) (F8BT:rrP3HT), applying the Variable Pump Intensity (VPI) and the Variable Stripe Length (VSL) methods. We demonstrate that, among all the spectral features affected by the presence of ASE, the most sensitive is the spectral linewidth and that the best way to estimate the ASE threshold is to determine the excitation density at the beginning of the line narrowing. We also show that the methods most frequently used in literature always overestimate the threshold up to more than one order of magnitude.

## 1. Introduction

Earliest discoveries of stimulated emission from conjugated polymers, dating back to 1992 [1] and 1996 [2], gave rise to intense studies on the optical gain and lasing properties of these materials, aiming to develop optically and electrically pumped lasers. Conjugated molecules show unique properties such as wide chemical flexibility, high photoluminescence quantum efficiency (PLQE), high stimulated emission cross section and electrical conductivity [3,4,5,6,7,8,9,10,11,12]. Moreover, organic materials can be easily processed: they can be deposited from solution through simple techniques such as spin coating [13], drop casting [14], ink-jet printing [15,16], dip coating [17], solution shearing [18] and blade coating [19].

To date, optical gain and optically pumped lasing have been demonstrated in many families of conjugated molecules and with a wide range on different resonators [20,21] and a first indication of lasing effect under electrical pumping has been recently reported [22]. In particular the organic systems showing optical gain can be divided in the two big families of neat films or blends of active molecules [2,4,5,6,7,8,9,10,11,12,23,24,25,26,27,28,29,30,31,32,33,34,35,36,37,38,39,40,41,42,43,44,45,46,47,48,49,50,51,52,53,54,55,56,57,58,59,60,61,62,63], most of the times polymeric, and of blends between inert polymers and small molecules [30,64,65,66,67,68,69,70,71,72,73].

The neat films have the advantage to preserve the charge mobility, and are thus potentially interesting for the development of laser diodes, while the blends with inert polymers allow to avoid quenching effects due to intermolecular aggregation, and are thus particularly interesting for applications to optically pumped lasers.

Overall the actual performances of organic lasers are still not high enough to allow commercial applications, thus stimulating further research to develop novel active materials.

A typical initial experiment for the characterization of a new material for laser applications is the quantification of its Amplified Spontaneous Emission (ASE) properties. The active material is deposited in the form of thin films on quartz or glass substrates, thus obtaining a planar asymmetric waveguide. The photoluminescence (PL) spectra are acquired as a function of the excitation density and, if the material shows optical gain, for high enough excitation density the spontaneous emission is amplified during the propagation along the waveguide, allowing to observe the appearance of an ASE band in the PL spectra.

In order to compare different active materials and to determine the best candidate for a potential laser implementation, the ASE threshold, representing the minimum excitation density which allows light amplification, is typically used. Materials that show low ASE threshold are considered good laser active materials, and vice-versa.

**Figure 1:**
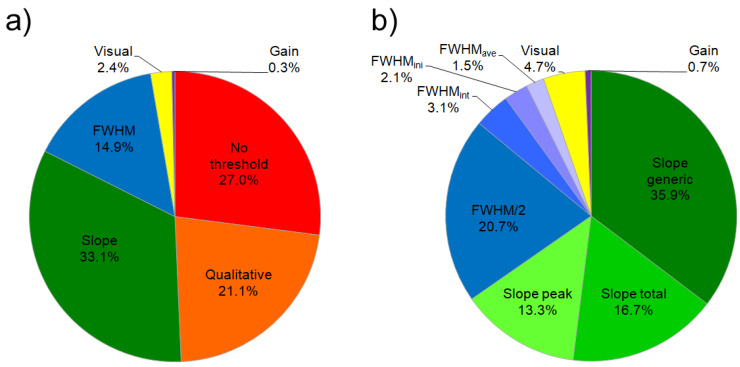
(**a**) Pie chart showing the percentage of papers on Amplified Spontaneous Emission (ASE) in polymers that quantify the ASE threshold in different ways. (**b**) Pie chart of the distribution of the main methods to quantify the ASE threshold.

Despite the importance of the ASE threshold value for the quantification of light amplification properties of materials and for their comparison, to date there is no consensus on the correct way to extract this parameter from the excitation density dependence of the PL spectra (Variable Pump Intensity, VPI, method). In order to have a complete picture of the current state of the art we focused our attention to experiments on the ASE properties of active polymers and we investigated 297 papers published to date, found by a research on Scopus with the string “Amplified Spontaneous Emission polymer” within “Article title, abstract, keywords” and written in English (see Figure 1) and manually removing the papers on inert polymer-dye blends and on non organic materials. The first interesting result (see Figure 1a) is that in 27.0% of the papers on ASE in polymers actually there is not any estimate of the threshold, while in 21.1% of the papers only qualitative values are provided. This means that overall in about one half of the papers the ASE threshold is not present at all, or in the best case only a rough evaluation is given. Focusing the attention on the 51.9% of the papers in which a procedure to determine the threshold is described, we identified the use of 9 different methods (see Figure 1b).

Most of these methods exploit the differences in the intensity increase with the excitation density and in the linewidth between the spontaneous emission and the ASE.

In particular, ASE is characterized by an intensity increase with the excitation density stronger than the spontaneous emission and its spectral lineshape is typically much narrower. Thus, the transition between the excitation density regime dominated by the the spontaneous emission and the one in which ASE prevails can be identified by observing the variation of the emission intensity and of the spectral linewidth as a function of the excitation density.

About 65.9% percent of the papers thus determine the ASE threshold as the excitation density at which the plot of the emission intensity vs. the excitation density shows a slope increase [23,24,25,26,27,28,29], looking at the PL total intensity (area under the PL spectrum) in 16.7% of the papers, at the ASE peak intensity in the 13.3%, or without specifying which "intensity" has been investigated in the majority of the cases (35.9%).

About 27.4% percent of the papers instead find out the ASE threshold from the excitation density dependence of the spectral linewidth, by considering the peak Full Width at Half Maximum (FWHM), that typically shows a constant value at low excitation density, when only spontaneous emission is present, a narrowing when ASE sets in and a further constant lower value when ASE dominates. Several different criteria are used, like the excitation density at which the FWHM attains one half of the value it has at low density [33,35,36,37,38] (FWHM/2, used in 20.7% of the papers), starts to decrease (even if without a clear definition) [39,40,41,42,43] (FWHM${}_{\mathrm{nar}}$, 2.1%), reaches the average value between the values assumed at low and at high excitation density [44,45] (FWHM${}_{\mathrm{ave}}$, 1.5%) or as the excitation density at which the extrapolation of the data representing the FWHM decrease takes the value corresponding to the low excitation density [24,46] (FWHM${}_{\mathrm{cros}}$, 3.1%).

A different approach exploits the lineshape variation due to the appearance of the ASE band, identifying the ASE threshold as the minimum excitation density at which the ASE band becomes visible [49,50,51,52,53,54,55,56] (Visual, 4.7%).

A last method, apparently more rigorous but also less diffused, defines the ASE threshold as the energy density at which the net gain of the active medium becomes zero, similarly to what happens in a laser cavity [74] (gain, 0.7%).

Even if all these methods are based on some effect related to the appearance of the ASE, the different criteria and experimental methods obviously affect the inferred values, making almost impossible any meaningful comparison of the obtained thresholds and, more importantly, opening the problem of understanding which is the most correct way to determine the ASE threshold value.

In this paper we report on a detailed quantitative comparison between all the main methods to determine ASE threshold, in order to quantify the dependence on the used method and to determine the best one in terms of reliability of the ASE value and of ease of application.

Our analysis is performed on thin films of three different conjugated polymers whose emission covers the entire visible range, namely poly(9,9-dioctylfluorene) (PFO), poly(9,9-dioctylfluorene-cobenzothiadiazole) (F8BT) and a blend of F8BT and regio regular Poly(3-hexylthiophene) (rrP3HT) (F8BT:rrP3HT). These polymers show good solubility in standard organic solvent, good film forming properties and efficient ASE [12,36,38,41,45,55], and can be thus considered good prototype materials for homopolymers (PFO), copolymers (F8BT) and host guest blends (F8BT:rrP3HT).

We demonstrate that, for all the samples, the lowest value of the ASE threshold is obtained by determining the excitation density at which the FWHM of the PL emission peak starts to decrease. We also demonstrate that the two most widespread methods, which consider the output intensity slope variation or the FWHM halving, used in about 86.6% of the published papers that quantify the threshold, provide similar thresholds, but these values are between 2 and 14 times higher than the best threshold estimate. These results are evidence that, among all the spectral features depending on the ASE appearance, the most sensitive one is the FWHM decrease, that is used in only 2.1% of the papers. On the contrary our results suggest that in about 98% of the papers on ASE properties of conjugated polymers the threshold values are overestimated, up to more than one order of magnitude. These results are expected to be of general validity and can provide a useful starting base for the correct quantification of the ASE threshold in polymeric active waveguides.

## 2. Results

All the investigated methods to determine the ASE threshold are based on the excitation density dependence of the PL spectra. In order to probe all the samples in comparable pumping conditions we initially evaluated the minimum excitation density necessary to observe a PL lineshape variation, due to the ASE band appearance. This excitation density value defines the first ASE threshold estimation (called visual in the following) and allows to fix the excitation density range to perform the VPI measurements. For all the samples we acquired 25 PL spectra at different excitation densities between about 1/10 and about 10 times the visual threshold.

**Figure 2:**
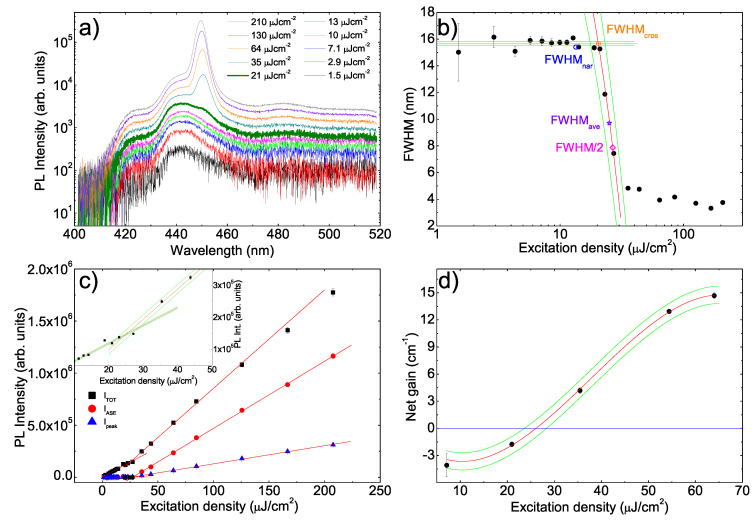
(**a**) Excitation density dependence of the photoluminescence (PL) spectra of the poly(9,9-dioctylfluorene) (PFO) sample. The thicker line evidences the first spectrum in which the lineshape is modified by the ASE presence. Only 10 spectra of the 25 acquired ones are shown for clarity. (**b**) Excitation density dependence of the PL spectra Full Width at Half Maximum (FWHM). The red lines are the best fit curves and the green lines the limits of the uncertainty range. The colored empty symbols represent the 4 threshold values extracted by ${\mathrm{FWHM}}_{\mathrm{nar}}$, ${\mathrm{FWHM}}_{\mathrm{cros}}$, FWHM/2 and ${\mathrm{FWHM}}_{\mathrm{ave}}$ methods. (**c**) Excitation density dependence of the integrated PL intensity (I${}_{\mathrm{TOT}}$) of the integrated ASE intensity (I${}_{\mathrm{ASE}}$) and of the intensity at the ASE band peak wavelength (I${}_{\mathrm{peak}}$). The red lines are the best fit curves. Inset: magnification of the I${}_{\mathrm{TOT}}$ data evidencing the crossing of the best fit lines used to determine the threshold and the uncertainty bands. (**d**) Excitation density dependence of the net gain. The blue line evidences the zero.

The PL spectra of the PFO sample show (see Figure 2a), at low excitation density, the typical spontaneous emission spectrum of the PFO glassy phase with the 0–0 transition peak at about 425 nm, followed by 0–1 and 0–2 vibronic replicas at about 443 and 480 nm [55]. As the excitation density increases the lineshape is unchanged, up to an excitation density value of 21 $\mu {\mathrm{Jcm}}^{-2}$ at which the spectrum shows a shoulder at about 450 nm (visual threshold). At higher excitation densities a clear ASE band peaked at 450 nm is observed, due to amplification of the 0–1 spontaneous emission, progressively dominating the PL spectra.

A progressive blue shift of the ASE band is observed with the excitation density, already observed in other samples of organic waveguides showing ASE, and typically ascribed to the competition between ASE and intermolecular energy migration within the disordered density of states [75,76,77].

The ASE band shows an intensity growth stronger than the spontaneous emission one and a lower linewidth, thus allowing to exploit the excitation density dependence of the emission intensity and of the spectral linewidth in order to determine the ASE threshold.

Concerning the line narrowing we observe that the FWHM is typically used in order to quantify the spectral linewidth. The obtained values (see Supplementary Materials for details) show (see Figure 2b) an almost constant value of about 16 nm up to about 14 $\mu J\;{\mathrm{cm}}^{-2}$, a progressive decrease up to about 35 $\mu J\;{\mathrm{cm}}^{-2}$ and a constant value of about 4 nm at higher excitation densities. In order to have a quantitative description of this behavior, we performed a best fit with a constant function up to 14 $\mu J\;{\mathrm{cm}}^{-2}$ and with a linear decrease between 21 $\mu J\;{\mathrm{cm}}^{-2}$ and $27\;\mu J\;{\mathrm{cm}}^{-2}$.

From the best fit curves (see Figure 2b) we determined the threshold as the excitation density at which:

- the linewidth reaches one half of its constant value at low excitation density (FWHM/2);
- the linewidth reaches the average value between the high one at low excitation density and the low one at high excitation density (${\mathrm{FWHM}}_{\mathrm{ave}}$);
- the linewidth starts to decrease (${\mathrm{FWHM}}_{\mathrm{nar}}$);
- the extrapolations of the two best fit lines cross (${\mathrm{FWHM}}_{\mathrm{cros}}$).

The intervals of the best fit functions have been determined by changing the constant term in the fit function within one standard deviation, as obtained by the fit procedure. These intervals were then used to estimate the uncertainty of the threshold values.

Concerning the ${\mathrm{FWHM}}_{\mathrm{nar}}$ threshold we considered the first point below the lower constant error line, representing the first point which deviates from the best line fit for more than one standard deviation. We thus estimated the threshold as the average between the excitation densities of this point and of the one immediately before, using their semidispersion as maximum error and converting it to statistical error. The obtained values, also reported in Table 1, are $\left(26.7\pm 1.6\right)\;\mu J\;{\mathrm{cm}}^{-2}$, $\left(25.0\pm 1.6\right)\;\mu J\;{\mathrm{cm}}^{-2}$, $\left(20.4\pm 1.7\right)\mu J\;{\mathrm{cm}}^{-2}$ and $\left(13.4\pm 0.4\right)\mu J\;{\mathrm{cm}}^{-2}$ for FWHM/2, ${\mathrm{FWHM}}_{\mathrm{ave}}$, ${\mathrm{FWHM}}_{\mathrm{cros}}$ and ${\mathrm{FWHM}}_{\mathrm{nar}}$, respectively.

The ASE threshold has been also determined exploiting the slope increase, due to the ASE presence, of the intensity raise with the excitation density (see Figure 2c). In this case we performed two different linear fits for the data before and after the slope increase, thus determining the threshold as the excitation density at which the two best fit lines cross. Three different values have been determined from the data of the total integrated intensity ($I_{\mathrm{TOT}}$), of the peak intensity at the ASE peak wavelength ($I_{\mathrm{peak}}$) and of the total integrated intensity of the ASE band (see Supplementary Materials for the details of the procedure used to separate the integrated ASE intensity from the total integrated intensity) ($I_{\mathrm{ASE}}$), namely $\left(24.8\pm 1.5\right)\;\mu J\;{\mathrm{cm}}^{-2}$, $\left(27.22\pm 0.51\right)\;\mu J\;{\mathrm{cm}}^{-2}$ and $\left(27.8\pm 1.1\right)\;\mu J\;{\mathrm{cm}}^{-2}$, respectively from $I_{\mathrm{TOT}}$, $I_{\mathrm{ASE}}$ and $I_{\mathrm{peak}}$ plots (Table 1).

Finally we determined the waveguide net gain by measuring the PL spectra at fixed excitation density and with stripe length varied between 0 and 4 mm in steps of 0.1 mm (VSL method). The measurements have been performed at five different excitation densities: below, close to and above the visual ASE threshold.

Assuming uniform gain along the stripe, the PL intensity dependence on the stripe length is given by [78]:

(1) $$I\left(\lambda ,g^{\prime },l\right)=\frac{I_{0}\left(\lambda \right)}{g^{\prime }\left(\lambda \right)}\left[e^{g^{\prime }\left(\lambda \right)l}-1\right]$$

where *l* is the stripe length, *g’* is the net optical gain coefficient and $I_{0}$ represents the spontaneous emission intensity per unity length. The experimental data (see Figure S2b) show an almost exponential increase with the stripe length, followed by a slower growth at high values of the stripe length. This effect is related to gain saturation, that is not included in the model. For this reason the data affected by gain saturation have been excluded in the fitting.

At each excitation density the net gain value $g^{\prime }$, given by the difference between the gain and the losses ($g^{\prime }=g-\alpha$), has been obtained from the best fit with equation 1 of the intensity increase with the stripe length, at each wavelength (see Figure S2b). The best fit values of the net gain $g^{\prime }$ at the ASE peak wavelength of 450 nm at all the investigated excitation densities show (see Figure 2d) a progressive increase with the excitation density, starting from negative values for the two lowest excitation densities. This dependence has been reproduced with a third degree polynomial fit, obtaining a last estimate of the ASE threshold of $\left(26.1\pm 1.4\right)\;\mu J\;{\mathrm{cm}}^{-2}$, by determining the excitation density at which the net gain becomes 0, evidencing that the gain compensates the losses.

**Figure 3:**
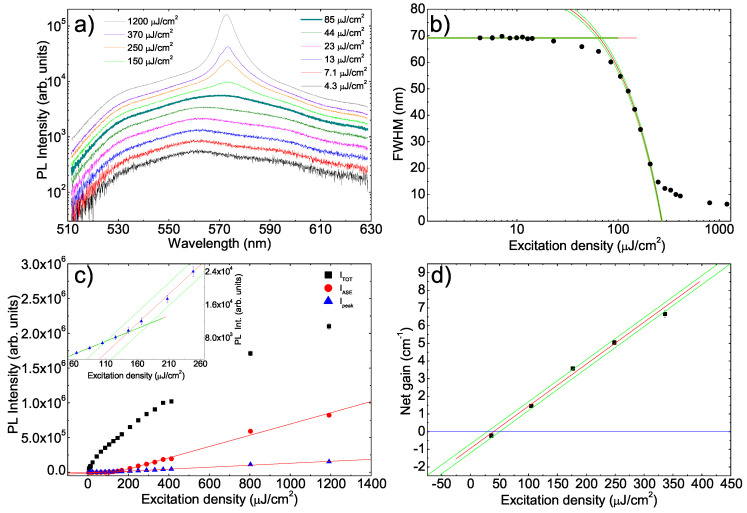
(**a**) Excitation density dependence of the PL spectra of the F8BT sample. The thicker line evidences the first spectrum in which the lineshape is modified by the ASE presence. Only 10 spectra of the 25 acquired ones are shown for clarity. (**b**) Excitation density dependence of the PL spectra FWHM. The red lines are the best fit curves and the green lines the limits of the uncertainty range. (**c**) Excitation density dependence of the integrated PL intensity (I${}_{\mathrm{TOT}}$) of the integrated ASE intensity (I${}_{\mathrm{ASE}}$) and of the intensity at the ASE band peak wavelength (I${}_{\mathrm{peak}}$). The red lines are the best fit curves. Inset: magnification of the I${}_{\mathrm{peak}}$ data evidencing the crossing of the best fit lines used to determine the threshold and the uncertainty bands. (**d**) Excitation density dependence of the net gain. The blue line evidences the zero.

A similar experiment has been performed on the F8BT film. The PL spectra show, at low excitation density (see Figure 3a), the presence of a single broad band, with a shoulder at about 536 nm, a main peak at about 561 nm and a further shoulder at about 593 nm due to the 0–0, 0–1 and 0–2 transitions, respectively. As the excitation density increases the lineshape does not change up to 85 $\mu J\;{\mathrm{cm}}^{-2}$ (visual threshold) at which a shoulder at about 573 nm starts to be visible. At higher excitation density a clear ASE band appears, with a peak wavelength of 573 nm [12,58].

In order to determine the ASE threshold from the spectral line narrowing we determined, from all the acquired spectra, the PL FWHM (see Figure 3b). The obtained results show a more gradual transition from the high value below the ASE threshold to the low value well above the threshold with respect to PFO, evidencing a slower increase of the ASE relative contribution to the total emission. From the best fit with a constant up to 11 $\mu {\mathrm{Jcm}}^{-2}$ and a linear decrease between 100 $\mu J\;{\mathrm{cm}}^{-2}$ and 210 $\mu J\;{\mathrm{cm}}^{-2}$ we determined the threshold values: ${\mathrm{FWHM}}_{\mathrm{nar}}$ = $\left(12.03\pm 0.41\right)\mu J\;{\mathrm{cm}}^{-2}$, ${\mathrm{FWHM}}_{\mathrm{cros}}$ = (64.9±2.4) $\mu J\;{\mathrm{cm}}^{-2}$, FWHM/2=$\left(168.0\;\pm \;2.0\right)\;\mu J\;{\mathrm{cm}}^{-2}$ and ${\mathrm{FWHM}}_{\mathrm{ave}}$ = $\left(158.0\pm 2.0\right)\;\mu J\;{\mathrm{cm}}^{-2}$ (see also Table 1).

Concerning the excitation density dependence of the emission intensity we observe that the integrated ASE intensity and the ASE peak intensity show the typical kink related to the ASE appearance (see Figure 3c). This behavior is instead not observed for the total integrated emission intensity, that shows a gradual increase in all the investigated range, despite the presence of the ASE contribution (above the threshold). In order to understand this anomalous result we observe that, at the highest explored excitation density of 1200 $\mu J\;{\mathrm{cm}}^{-2}$, the integrated ASE intensity is about only 0.39 times the total intensity. This clearly evidences that, despite the progressive increase of the ASE contribution, even at the highest excitation density the total emission is mainly due to the spontaneous emission that has a clearly lower peak intensity, but also a much larger linewidth. Thus, the appearance of the ASE band in the spectra does not result in a clear variation of the integrated intensity growth, hidden by the dominating contribution of the spontaneous emission.

The ASE threshold has been thus determined only from the best fit with linear functions of the data of the integrated ASE intensity and of the ASE peak intensity, obtaining a value of $\left(142\pm 15\right)\;\mu J\;{\mathrm{cm}}^{-2}$ and $\left(145\pm 20\right)\;\mu J\;{\mathrm{cm}}^{-2}$, respectively.

The last ASE estimate has been obtained from the excitation density dependence of the net gain at the ASE peak wavelength of 573 nm (see Figure 3d), showing an almost linear increase with the excitation density. The excitation density of zero net gain is $\left(39.5\pm 6.1\right)\mu J\;{\mathrm{cm}}^{-2}$.

**Figure 4:**
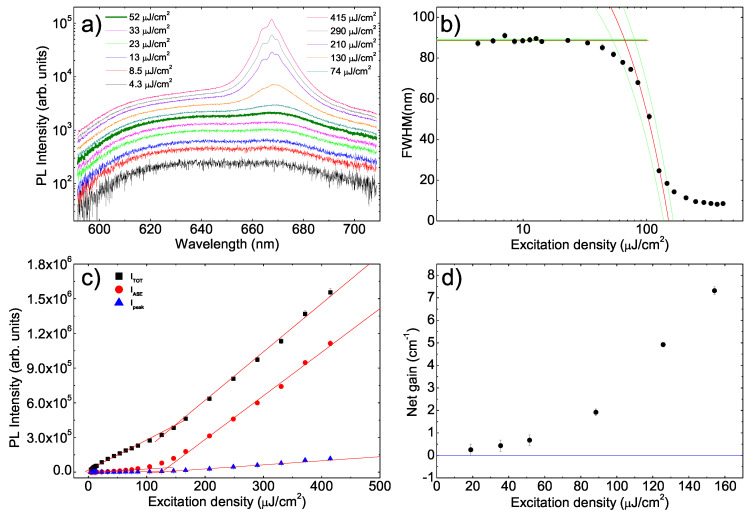
(**a**) Excitation density dependence of the PL spectra of the F8BT:rrP3HT sample. The thicker line evidences the first spectrum in which the lineshape is modified by the ASE presence. Only 11 spectra of the 25 acquired ones are shown for clarity. (**b**) Excitation density dependence of the PL spectra FWHM. The red lines are the best fit curves and the green lines are the limits of the uncertainty range. (**c**) Excitation density dependence of the integrated PL intensity (I${}_{\mathrm{TOT}}$) of the integrated ASE intensity (I${}_{\mathrm{ASE}}$) and of the intensity at the ASE band peak wavelength (I${}_{\mathrm{peak}}$). The red lines are the best fit curves. (**d**) Excitation density dependence of the net gain showing the lack of negative values. The blue line evidences the zero.

The last investigated sample is the F8BT:rrP3HT blend film. The PL spectra show, below the ASE threshold, the typical linewidth of the rrP3HT emission (see Figure 4a), with a 0–0 line at about 640 nm and a 0–1 vibronic replica at about 666 nm. The absence of F8BT PL is due to the efficient F8BT→rrP3HT Förster Resonant Energy Transfer [36,38]. As the excitation density reaches 52 $\mu J\;{\mathrm{cm}}^{-2}$ the relative intensity of the 0–1 line starts to increase (visual ASE threshold) and, at higher excitation density, a clear ASE band peaked at about 668 nm is observed. In this sample the ASE band shows a weak modulation, well visible above 130 $\mu J\;{\mathrm{cm}}^{-2}$, likely due to random lasing assisted by scattering from morphological irregularities in the film [59,60,61,62].This attribution has been confirmed by SEM measurements showing (see Figure S3c,d) the lack of thickness uniformity in the film. In particular the thickness is about 1.25μm close to the substrate edge, and progressively decreases to about 340 nm close to the film center. This effect is likely related to the deposition from a hot solution and to the solution temperature decrease during the spin coating, leading to a higher thickness toward the edges.

Similarly to the F8BT films, the spectral FWHM shows a gradual transition from the constant value of about 89 nm well below threshold to about 8.5 nm well above it (see Figure 4b). Concerning the emission intensity increase with the excitation density we observed a clear slope variation, related to the ASE appearance, in the total integrated intensity, the ASE integrated intensity and the ASE peak intensity (see Figure 4c). Following the already described procedures we thus estimated the ASE threshold values reported in Table 1.

On the contrary, the excitation density dependence of the net gain at the ASE peak wavelength (see Figure 4d) shows a progressive increase with the excitation density, but it never shows negative gain values, even at the lowest input energy density that is 1.5 times below the lowest quantitative estimate of the ASE threshold and about 3 times below the visual ASE threshold. The lack of transition between negative and positive values of the net gain prevented an evaluation of the ASE threshold from the $g^{\prime }=0$ condition.

## 3. Discussion

All the obtained ASE values are determined by exploiting the effects of the ASE appearance on several spectral features, like linewidth, lineshape and output intensity, or by relating the ASE to the presence of positive net gain. In this section we will compare the values extracted from the all the investigated methods in order to determine the most reliable one and thus the best method to correctly quantify the ASE threshold.

As a preliminary step we observe that the ASE threshold is the minimum value of the excitation density that induces the ASE presence. By definition of minimum all the experimental values, determining the excitation density at which some effect related to ASE becomes observable, cannot be lower than the real threshold. For this reason, among all the experimental values, the lowest value will be the one closest to the real threshold, and will thus provide the most reliable estimate of the threshold.

**Figure 5:**
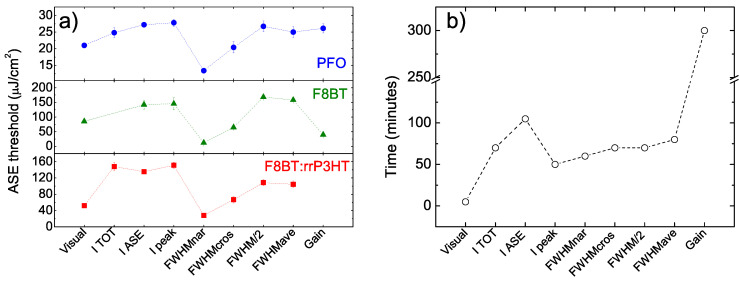
(**a**) ASE threshold values for PFO (top), F8BT (middle) and F8BT:rrP3HT (bottom). (**b**) Estimated working times for all the methods. All the lines are guides for the eyes.

The plot of the threshold values as a function of the used method (see Figure 5a) allows to observe a similar trend for all the three investigated polymers evidencing that the relative values extracted by different methods are mostly independent of the specific material.

In particular, for all the investigated samples, the lowest ASE threshold is always obtained by the FWHM${}_{\mathrm{nar}}$ method. This allows us to conclude that the quantity most sensitive to the ASE presence is the spectral linewidth and that the beginning of the line narrowing allows to determine the lowest threshold value. For this reason the method FWHM${}_{\mathrm{nar}}$ can be considered the best one for a reliable determination of the ASE threshold value.

It is important to observe that, rather surprisingly, the ASE threshold is determined by the FWHM${}_{\mathrm{nar}}$ method only in about 2.1% of the papers quantifying the ASE threshold, evidencing that in the remaining 97.9% the reported thresholds are systematically overestimated.

The sensitivity of the FWHM decrease to the ASE appearance can be clearly evidenced by observing that in all the samples the line narrowing is detectable at an excitation density much lower than the one that allows to observe a variation of the linewidth (given by the visual threshold). In particular the two samples with the highest relative ASE contribution to the emission, i.e., PFO and F8BT:rrP3HT, show a FWHM${}_{\mathrm{nar}}$ threshold about 2 times lower than the visual one, while in F8BT, that shows the lowest relative ASE intensity, the linewidth reduction starts at an excitation density 7 times smaller than the visual threshold.

Concerning the other methods the ones that provide ASE threshold closer to the FWHM${}_{\mathrm{nar}}$ are always the visual method and the ${\mathrm{FWHM}}_{\mathrm{cros}}$, both leading to similar ASE threshold values (with relative differences between almost negligible for PFO, and of about 30% for F8BT). This suggests that the excitation density determined by ${\mathrm{FWHM}}_{\mathrm{cros}}$ basically coincides with the one of ASE appearance in the spectra.

Moving to the most popular methods, we observe that the threshold values obtained with I${}_{\mathrm{tot}}$, I${}_{\mathrm{ASE}}$ and I${}_{\mathrm{peak}}$ are always comparable within about 10%. However these values are systematically much larger than the FWHM${}_{\mathrm{nar}}$ ones, with differences between about 2 times for PFO up to 12 times for F8BT.

The threshold values obtained by the FWHM/2 method are overall comparable with the ones extracted from the intensity slope increase, with relative differences between almost 0 for PFO and about 28% for F8BT:rrP3HT.

One should be aware that the methods based on the slope variation and the FWHM/2 one lead to a systematic overestimation of the threshold, that is dependent on the specific material and that can exceed one order of magnitude.

The threshold overestimation is intrinsic of the method definitions as both the intensity slope increase and the FWHM halving are obtained when the ASE contribution starts to dominate the emission, and not when ASE emission appears.

Finally we observe that the threshold values obtained from the $g^{\prime }=0$ condition are completely unpredictable and not reliable. This is due to the dependence of the gain values on the thickness uniformity; so irregularities result in a limited capability to determine the net gain with the accuracy (within fraction of cm${}^{-1}$) necessary to correctly find the excitation density of 0 net gain. Our samples clearly show this effect, as PFO is the sample with the highest thickness uniformity and shows a threshold value obtained from the gain value comparable to the ones obtained from the intensity and the FWHM halving. F8BT, presenting some thickness fluctuations, is instead characterized by a clearly reduced value obtained from the gain. Finally the F8BT:rrP3HT film, that is the worst sample in terms of uniformity, never exhibits negative gain values.

Another important aspect to consider in order to compare the different methods is their ease of use, that can be evaluated from an estimate of the total working time that they need (see the Supplementary Materials for details and Figure 5b).

In this case we observe that the visual method is largely the fastest one, needing just a few minutes, and it is the most suitable for the investigation of the ASE threshold uniformity across the sample in short times. All the other methods based on VPI experiments are instead roughly comparable in term of working times, strongly suggesting that FWHM${}_{\mathrm{nar}}$ provides overall the best choice to combine correct values and reasonable measurement times. The only foresight in order to be able to determine the beginning of the line narrowing with a small error bar is to acquire a reasonable number of spectra at excitation densities below the visual threshold one. In our experiment the presence of 10 spectra below the visual threshold and starting from an excitation density about 10 times below it allowed to determine the FWHM${}_{\mathrm{nar}}$ threshold with a relative uncertainty below 10% in all the samples.

Moreover, by looking at the three methods based on the intensity growth analysis we suggest I${}_{\mathrm{peak}}$ as the best choice, as it allows to get a threshold value comparable with I${}_{\mathrm{TOT}}$ and I${}_{\mathrm{ASE}}$, but in clearly shorter times.

Finally, also working time considerations strongly suggest to avoid the use of gain measurements to quantify the threshold as, beyond the risk of completely unreliable values, the necessary time to get the threshold values are at least 4 times higher than the FWHM${}_{\mathrm{nar}}$ ones. It is also relevant to evidence that this time has been estimated for the determination of the gain at a single wavelength. In our experiment the maximum gain wavelength has been determined from the gain spectra including values at 75 different values around the ASE peak wavelength, considerably increasing the time to obtain each reported value.

## 4. Materials and Methods

### 4.1. Sample Preparation

PFO and F8BT were provided by ADS dyes (Canada) while rrP3HT was provided by Sigma Aldrich (now Merck KGaA, Germany). All the polymers have been used as received.

All the samples have been realized by spin coating from 15 mg/mL toluene solutions, with a rotation speed of 500 rpm for 5 s and 2500 rpm for 2 min, on glass substrates.

The PFO solution has been heated at 50${}^{\circ }$ for few minutes, in order to avoid the formation of the β-phase [55,63].

The F8BT:rrP3HT blend has been prepared by mixing two 15 mg/mL toluene solutions of the individual materials with a relative concentration of 80:20 by weight, chosen in order to minimize ASE threshold [38]. In order to avoid rrP3HT aggregation the final solution has been heated at about $60^{\circ }$ and the spin coating has been done from the hot solution [36,38].

### 4.2. VPI and VSL Measurements

All the samples have been excited by a LTB MNL 100 Nitrogen laser, delivering 3 ns pulses with a repetition rate of 10 Hz and a wavelength of 337 nm. The laser beam has been focused on the sample by a cylindrical lens, obtaining a rectangular pump stripe with 80 μm width. The stripe length has been varied through a micrometric slit up to 4 mm. The pump stripe has been placed at the edge of the film and the edge emitted radiation has been collected by a lens system coupled with an optical fiber connected to a computer-controlled Acton 750 spectrometer, and detected by an Andor Peltier cooled CCD. The spectral resolution was 0.5 nm. All the measurements have been performed at room temperature and, in order to avoid photodegradation effects, in vacuum (10${}^{-2}$ mbar).

The VPI measurements have been performed by keeping the stripe length fixed at 4 mm and by changing the excitation density with a continuously variable neutral filter. As a first step in the measurements we determined the excitation density at which the ASE band starts to be visible in the spectra by acquiring the PL spectra in real time while progressively increasing the excitation density. The VPI measurements have been then performed at 25 different excitation density values from 1/10 to 10 times this value.

The VLS measurements have been instead performed by keeping fixed the excitation density and by changing the pump stripe length from 0 mm to 4 mm in steps of 0.1 mm. The measurements have been performed at no less than 5 different values of the excitation density between 1/3 and 3 times the visual ASE threshold.

### 4.3. Thickness Measurements

The film thickness has been determined from Scanning Electron Microscopy (SEM) images in cross section. The images have been collected by a JEOL JSM-6480LV SEM, operated at 20 kV. In order to prevent charging effects the samples have been metalized by depositing a 10 nm-thick gold film on the surface by sputtering in Ar atmosphere at a pressure of 10${}^{-1}$ mbar, with a Quorum Technologies- Emitech K550x sputter coater.

## 5. Conclusions

In conclusion, we reported a quantitative comparison among various currently employed methods to determine the ASE threshold of organic active waveguides, in order to find the one that allows to determine the most accurate estimate of the ASE threshold.

We demonstrated that the spectral feature most sensitive to the ASE appearance is the spectral linewidth, and that the most reliable way to quantify the ASE threshold is to determine the excitation density at which the line narrowing starts. We also evidenced that the most common methods used in literature, determining the threshold from the slope variation of the intensity growth or from the FWHM halving, permit to determine the excitation regime at which the ASE starts to dominate the emission, but systematically overestimate the ASE threshold up to 14 times. Our results will be useful to correctly quantify the ASE threshold in polymeric waveguides, and to easily compare the ASE properties of different novel materials.

## Figures and Tables

**Table 1:** ASE threshold values obtained with all the methods and for all the investigated samples.

Method	ASE Threshold ($\mu J\;{\mathrm{cm}}^{-2}$)		
	PFO	F8BT	F8BT:rrP3HT
Visual	∼21	∼85	∼52
$I_{\mathrm{TOT}}$	24.8±1.5	N/A	148±10
$I_{\mathrm{ASE}}$	27.2±0.5	142±15	135.2±5.6
$I_{\mathrm{peak}}$	27.8±1.1	145±20	151.3±7.7
FWHM/2	26.7±1.6	167.9±2.0	108.6±7.8
${\mathrm{FWHM}}_{\mathrm{ave}}$	25.0±1.6	158.0±2.0	104.6±7.8
${\mathrm{FWHM}}_{\mathrm{cros}}$	20.4±1.7	64.9±2.4	66.5±7.9
${\mathrm{FWHM}}_{\mathrm{nar}}$	13.4±0.4	12.03±0.41	28.1±3.0
Gain	26.1±1.4	39.5±6.1	N/A

## Supplementary Materials

The following are available online, Figure S1: Example of the variation of the peak wavelength with the excitation density for the PFO film, Figure S2: a: Example of the determination of the ASE integrated intensity for the PFO film. b: Intensity increase with the stripe length for the PFO film and best fit line. Figure S3: SEM image in cross section of the investigatd samples, Table S1: Estimated working time in minutes for the determination of the ASE threshold values for each method.
