# Supplementary material for: Determination of the Best Empiric Method to Quantify the Amplified Spontaneous Emission Threshold in Polymeric Active Waveguides
Source: Molecules. 2020 Jun 30;25(13):2992. doi: 10.3390/molecules25132992 (PMC7411902; doi:10.3390/molecules25132992)
Supplement: Supplementary file 1 [file molecules-25-02992-s001.pdf]

Supplementary Materials: Determination of the Best Empiric Method to Quantify the Amplified Spontaneous Emission Threshold in Polymeric Active Waveguides

Stefania Milanese [1], Maria Luisa De Giorgi[1] and Marco Anni[1,*]

1. Determination of the spectral Full Width at Half Maximum

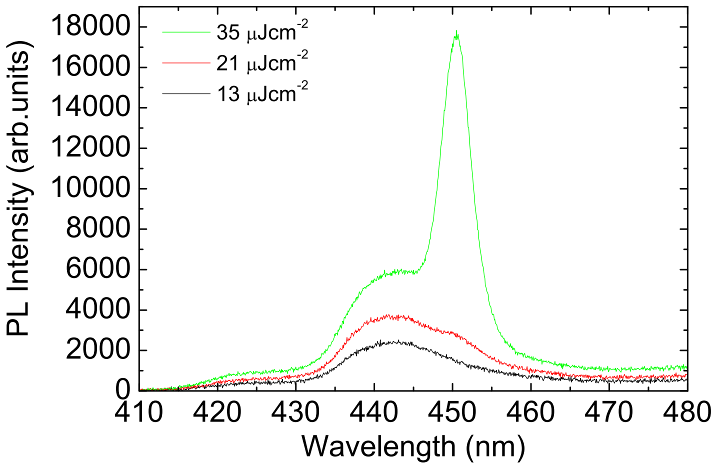

Figure S1. Example of the variation of the peak wavelength with the excitation density for the PFO film.

The FWHM has been determined, at each excitation density, as the spectral interval in which the emission intensity is above 50% of the peak one, detected at the wavelength of maximum emission. In order to obtain the FWHM value we took into account that the ASE band, as typical of materials showing light amplification, is at higher wavelength that the spontaneous emission peak. This result is due to the decrease of self absorption when the wavelength increase, leading to the best compromise between high gain and low losses at a wavelength higher than the spontaneous emission peak one.

At low excitation density, when ASE is not present, the spectral lineshape is independent of the excitation density and the peak wavelength is also constant. When the ASE appears in the spectra the wavelength of maximum emission changes, and the peak intensity has to be detected at the correct peak wavelength. For example, with reference to the PFO spectra shown in Fig. S1, we observe that the spectra at 13 μJcm^{-2} and 21 μJcm^{-2} have a linewidth determined by the 0-1 line one, thus we considered as peak wavelength 443 nm, while the spectrum at 35 μJcm^{-2} is already dominated by ASE, thus we considered as peak wavelength the ASE peak one (about 451 nm).

Considering that the emission spectra of polymers often show the contribution of the 0-0 line and of different vibronic replicas the FWHM below threshold will reflect the linewidth of the peak that generates ASE only if this peak also dominates the spontaneous emission af it is well resolved. Otherwise the FWHM values below the threshold will be affected by the contribution of different peaks.

For the samples investigated in this experiment we observe a well resolved vibronic progression only for PFO, with a spontaneous emission dominated by the 0-1 line, that also gives the ASE peak. In this case our procedure allows to determine the 0-1 FWHM in the whole excitation density range. For the other two samples the vibronic peaks are not resolved, thus leading to higher values of the FWHM at low excitation density. We anyway observe that this effect only affects the FHWM value below the

threshold, but it does not modify the FWHM excitation density dependence due to the narrower ASE band appearance, resulting in a FWHM decrease in all the samples.

2. Determination of the ASE integrated intensity and of the net gain

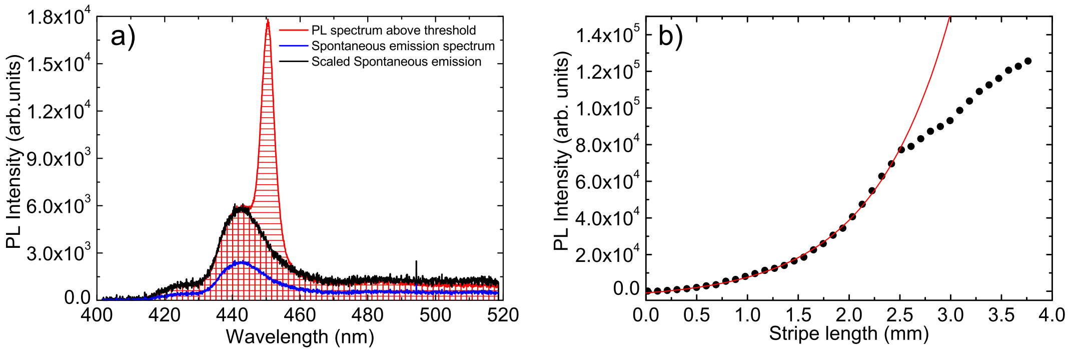

Figure S2. a: Example of the determination of the ASE integrated intensity for the PFO film. The ASE integrated intensity is evidenced by the region with horizontal red line pattern, while the contribution of the spontaneous emission to the total intensity is evidenced by the squared patterned region. b: Intensity increase with the stripe length for the PFO film (dots), the red line is the best fit function with Equation 1.

In order to determine the ASE integrated intensity at any given excitation density we plotted the corresponding PL spectrum (see red line in Fig. S2 a) and we initially determined the integrated emission intensity. We then separated the ASE contribution to the total integrated intensity by exploiting the lack of excitation density dependence of the spontaneous emission lineshape. We thus plotted a PL spectrum below the visual ASE threshold (blue line in Fig. S2 a) and we rescaled it in order to match the intensity of the spontaneous emission contribution to the total spectrum (black line in Fig. S2 a). We finally determined the total spontaneous emission intensity by numerical integration of the rescaled spectrum below threshold, and the integrated ASE intensity as difference between the total intensity and the spontaneous emission integrated intensity.

The net gain values have been instead determined taking care to exclude in the fitting procedure the data showing effects of gain saturation. For example the data shown in Fig. S2 b, relative to the PFO film, show an almost exponential increase up to a stripe length of 2.5 mm, while a sublinear increase is observed at higher values of the stripe length. In this case only the data up to a stripe length of 2.5 mm have been included in the fitting procedure.

3. Film thickness

The thickness of the investigated films has been determined by SEM measurements in cross section. The obtained images show (see Fig. S3 a and b) a uniform thickness for the PFO and F8BT films, of about 150 nm and 170 nm, respectively. On the contrary the thickness of the F8BT:rrP3HT film is clearly not uniform, with a lower thickness of about 340 nm in the central part and a progressive increase toward the sample edges up to about 1.25 μm (see Fig. S3 c and d).

4. Working time estimate

In order to estimate the time required to obtain the threshold value with the different methods we took into account the net time necessary for the measurements, for the data extraction and for the fitting procedures (see Tab. S1).

In particular we considered that the visual determination of the threshold simply requires to observe the PL spectra evolution while continuously increasing the excitation density, by running the acquisition CCD in real time. This procedure can be completed in about 1 minute and we typically

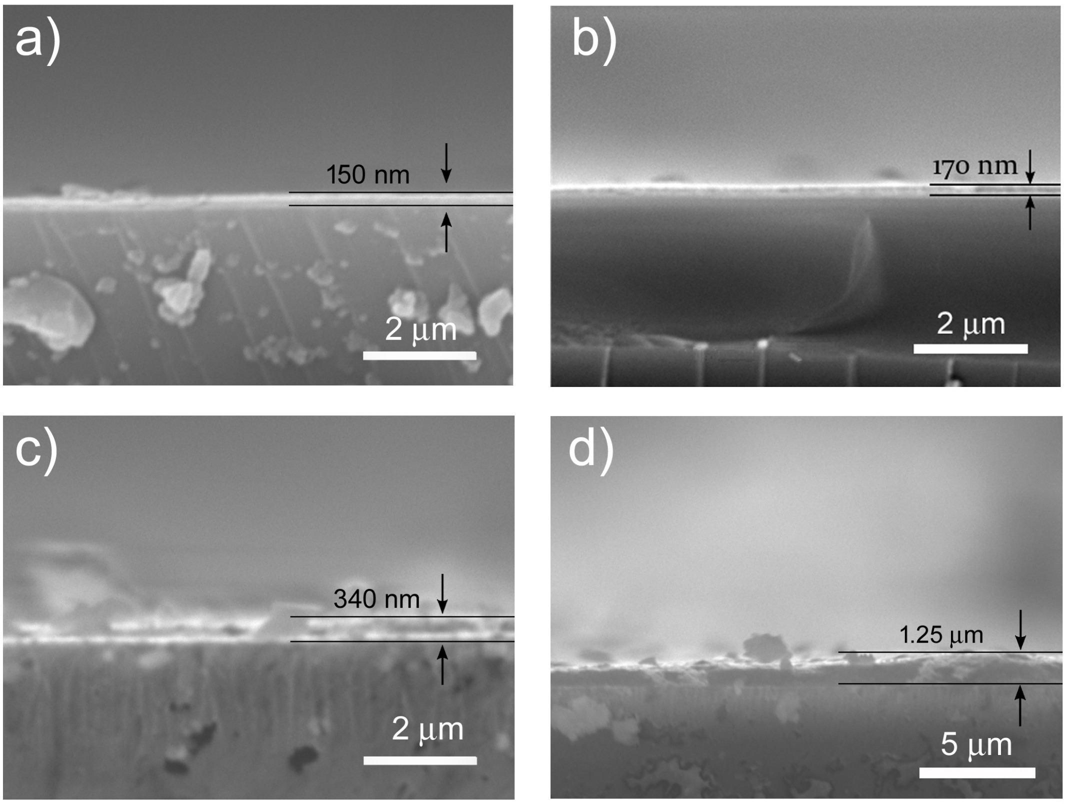

Figure S3. SEM image in cross section of a: the PFO film; b: the F8BT film; c: the central part of the F8BT:rrP3HT film and d: the F8BT:rrP3HT film close to the substrate edge, evidencing the progressive thickness increase.

repeat it 4-5 times, in order to safely determine the minimum excitation density that leads to a lineshape variation. We thus estimated that the determination of the visual threshold can take about 5 minutes.

For the other methods we first of all estimated the time to acquire each spectrum in about 1 minute, considering that the integration time is typically of few seconds, and that every measurement just needs to add the time for the setting of the acquisition parameter (excitation density in VPI experiments and stripe length in VSL ones) and for the file saving. The methods based on the VPI methods thus need about 25 minutes for the measurements. This limited time further evidences that the acquisition of a high number of spectra has a very limited impact on the total experiment working time and thus, on the contrary, there are not real reasons to limit the VPI experiment to the collection of a low number of spectra, as often done in literature.

The times for the VLS are instead much higher as we collected, at each excitation density, the PL spectra at 40 different values of the stripe length and we performed the whole experiment using at least 5 different values of the excitation density. Thus overall 200 measurements have been done for each sample.

Concerning the time for the data extraction we considered that the FWHM is estimated from the PL spectra plot. This work has been done with Origin 8 from OriginLab and it simply requires to select the data column, to press the plot command, to determine the PL peak value and then the FWHM. Overall, starting from fully imported data, this procedure requires about 1 minute for each value (thus overall 25 minutes).

The times needed for the calculation of the intensity value are instead different for I_{TOT}, I_{ASE} and I_{peak}. In particular each value of I_{TOT} is obtained by plotting the PL spectrum and numerically integrating it, requiring about 1 minute. The estimate of I_{ASE} is instead longer, as it requires the spectrum plotting and integration, but also the rescaling of the spectrum below threshold to match the spontaneous emission band, its integration and, finally, the subtraction of the two integrated intensities

Table S1. Estimated working time in minutes for the determination of the ASE threshold values for each method.

Method	Measurements	Data extraction	Best fit	Total
Visual	5	-	-	5
I_{TOT}	25	25	20	70
I_{ASE}	25	60	20	105
I_{peak}	25	5	20	50
$FWHM_{nar}$	25	25	10	60
$FWHM_{cros}$	25	25	20	70
FWHM/2	25	25	20	70
$FWHM_{ave}$	25	25	30	80
Gain	200	50	50	300

to obtain the integrated ASE intensity. Overall this procedure can be completed in a bit more than 2 minutes.

On the contrary the extraction of the I_{peak} value is much faster, as it simply requires to copy the intensity value at the ASE peak wavelength for each spectrum (we estimated about 10 seconds).

A similar assumption was made for the time needed to have the PL intensity at a given wavelength and stripe length in the VSL experiment.

Furthermore, we assumed that each best fit requires about 10 minutes.

We observe that the estimated time reported in the table for the VLS method corresponds to the determination of the net gain dependence on the excitation density at a single wavelength. In our experiment we determined the whole net gain spectrum by repeating the procedure for 75 different wavelength, thus increasing of 75 times the real working time.

Finally, we highlight that the total times in the last column of the table represent only a lower limit of the overall time taken by the experiment.
